# Cross-sectional study evaluating organizational climate, change commitment, and change efficacy for predicting family planning clinics’ success in increasing HIV counseling and testing in Mombasa, Kenya

**DOI:** 10.1371/journal.pgph.0005542

**Published:** 2025-12-31

**Authors:** Chipo Natasha Kwendakwema, McKenna C. Eastment, George Wanje, Barbra A. Richardson, Emily Mwaringa, Kenneth Sherr, Kishorchandra N. Mandaliya, Ruanne V. Barnabas, Walter Jaoko, Raymond Scott McClelland

**Affiliations:** 1 Department of Medicine, University of Washington, Seattle, Washington, United States of America; 2 Department of Global Health, University of Washington, Seattle, Washington, United States of America; 3 Department of Epidemiology, University of Washington, Seattle, Washington, United States of America; 4 Department of Biostatistics, University of Washington, Seattle, Washington, United States of America; 5 Vaccine and Infectious Disease Division, Fred Hutchinson Cancer Center, Seattle, Washington, United States of America; 6 Mombasa County Department of Health, Mombasa, Kenya; 7 Division of Infectious Diseases, Department of Medicine, Massachusetts General Hospital, Harvard Medical School, Boston, Massachusetts, United States of America; 8 Medical Microbiology and Immunology, University of Nairobi, Nairobi, Kenya; McGill University, CANADA

## Abstract

Increasing HIV testing and counselling (HTC) is a first step to reducing HIV transmission. Implementing HTC in family planning (FP) clinics has been proposed to increase HIV testing coverage in at-risk populations. The Systems Analysis and Improvement Approach (SAIA) was used to improve HTC rates in FP clinics in Mombasa, Kenya. This hypothesis-generating exploratory analysis evaluated the associations between organizational climate characteristics, organizational readiness for implementing change, and successful implementation of HTC. Surveys were conducted with clinic managers and staff from FP clinics implementing SAIA to increase HTC. Likert-style questions were used to characterize organizational climate metrics and organizational readiness for implementing change (ORIC). Linear regression was performed to examine the association between organizational climate metrics, ORIC domains, and two FP client outcomes: 1) percentage of clients receiving pre-HIV test counseling, and 2) percentage of clients tested for HIV. Eleven clinic staff and 10 clinic managers completed the surveys. For clinic staff, higher innovation and flexibility scores were associated with higher change commitment (β = 0.20, CI 0.09-0.31, p = 0.001) and change efficacy (β = 0.17, CI 0.07-0.26, p = 0.002). Higher clinic manager scores for innovation and flexibility were associated with a higher change commitment (β = 0.44, CI 0.04-0.84, p = 0.03). Additionally, clinic managers’ scores for management support (β = 0.25, CI 0.06-0.45, p = 0.01), commitment to facility (β = 0.78, CI 0.60-0.96, p = 0.001), and relative priority (β = 0.24, CI 0.08-0.39, p = 0.004) were positively associated with higher change commitment and change efficacy. In contrast, clinic managers’ scores for tradition were negatively associated with change commitment (β = -0.38, CI -0.75-0.01, p = 0.05). Clinic staff perceptions of management support were positively associated with the proportion of clients counseled for HIV testing (β = 1.20, CI 0.08-2.32, p = 0.04). Support from leadership and innovation/flexibility are important predictors of change commitment and change efficacy. Strong management support may increase the likelihood of successful implementation of SAIA to improve HTC.

## Introduction

Human immunodeficiency virus (HIV) is an important cause of morbidity and mortality in sub-Saharan Africa, with the highest burden of disease in women of childbearing age [1]. To combat the HIV epidemic, the Joint United Nations Programme on HIV/AIDS has proposed fast-track targets for 2025 that include 95% of people who are seropositive being aware of their HIV status, 95% of people who are living with HIV being on treatment, and 95% of people on treatment being virally suppressed [1]. Of these targets, the first 95 may be the most difficult to reach due to the substantial resources needed to identify at-risk populations, disseminate information, and acquire supplies [2,3]. Integration of HIV testing into routine family planning (FP) services has been proposed by the World Health Organization and adopted by many countries as a strategy to increase the proportion of people who know their HIV status [3]. However, there have been mixed results of integrating HIV testing and counseling (HTC) into FP clinics in the existing literature [4].

Kenya is a high-prevalence country with an estimated 880,000 women over age 15 living with HIV in 2022 [5]. Kenya’s National AIDS and STD Control Program first outlined goals for implementing HIV testing and counseling in FP clinics in 2008 [6]. Current national guidelines mandate that HTC services be available at all FP clinics [7]. However, data from FP clinics in Kenya suggests that HTC is still performed infrequently due in part to a variety of health systems barriers, including healthcare providers not recommending testing [8]. Given the high prevalence of HIV in sub-Saharan Africa, reaching HTC goals globally depends on progress in this region.

Implementation research can facilitate the initiation and uptake of evidence-based practices. The Systems Analysis and Improvement Approach (SAIA) is a multicomponent implementation strategy that iteratively uses a five-step cycle to identify modifiable areas for improvement and test contextually appropriate solutions [9]. SAIA has been used to improve implementation of complex multi-step evidence-based interventions in healthcare settings in Africa including prevention of mother-to-child transmission of HIV, hypertension diagnosis and care, and pediatric and adolescent HIV care [10–12]. SAIA has also been used to improve performance of HTC in FP clinics in Mombasa County, Kenya in a cluster randomized controlled trial (RCT) which is the basis of this analysis [13].

Organizational readiness for change, defined as the degree to which members of an organization are psychologically and behaviorally prepared to implement change, is an important precursor to successful institutional change [14,15]. Furthermore, in the setting of HTC, providers are often the ones to recommend HIV testing, and clinic managers are in charge of procuring commodities and administering policies and guidelines. For these reasons, it is important to understand clinic staff and managers’ readiness for change in this setting. Various tools and assessments have been developed to assess organizational readiness, including the Organizational Readiness for Implementing Change (ORIC) tool [16]. The ORIC tool is a psychometrically validated assessment with high reliability, construct validity, and internal consistently in different settings. This tool evaluates two facets of readiness: change commitment, which describes organizational members’ collective resolve to implement change, and change efficacy, which describes their belief in their capability to do so [16]. The ORIC tool has been used in various healthcare settings in high-resource environments to assess organizational readiness for change [17–21], though there have been fewer applications of the ORIC assessment in Africa [22,23]. In addition to the organizational readiness for change, organizational climate is another important construct that may be associated with the success of implementation of interventions. Organizational climate refers to the characteristics of a particular setting and a particular intervention in relation to management support, relative priority, commitment, communication within the facility, tradition (what has historically been done), innovation and flexibility to make changes, the effort that it takes to do something, and the level of support from supervisors.

This hypothesis-generating study aimed to assess organizational climate and ORIC domain scores for FP clinic staff and managers, and to examine whether features of the FP clinic organizational climate were associated with change commitment and change efficacy to implement HTC. The study also assessed whether features of organizational climate, change commitment, and change efficacy were predictive of FP clinics’ performance in HTC after implementing SAIA in Mombasa County, Kenya.

## Methods

We previously conducted a cluster randomized trial in 24 FP clinics in Mombasa County, Kenya. Detailed procedures and results of the RCT have been published (clinicaltrials.gov registration NCT02994355) [13]. Clinics were randomized 1:1 to implementing SAIA versus usual procedures. After randomization, clinic staff from intervention clinics attended a full day SAIA training. SAIA intervention cycles were repeated monthly over 12 months from December 2018 to November 2019. Study staff collected information on clinic characteristics at baseline with clinic managers. HIV testing and counselling data were collected monthly for the 12-month RCT.

**Fig 1:**
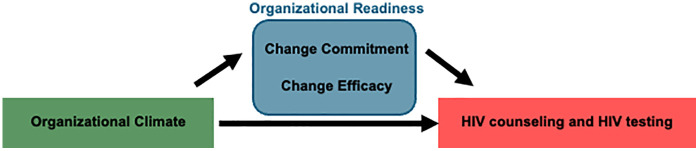
Conceptual model that outlines the hypothesized relationships between organizational climate, organizational readiness (change commitment and change efficacy), and the study outcomes of HIV counseling and HIV testing.

The present cross-sectional analysis focused on clinics in the intervention arm of the RCT. Structured face-to-face surveys were administered by research staff with one staff member involved in FP service delivery and the SAIA intervention and one manager from each intervention FP clinic during the second half of the trial. Surveys were administered in English, an official language in Kenya that is used widely in healthcare. The survey was administered separate from the SAIA RCT visits. Participants were eligible to participate in the survey if they were aged 18 years and older. All FP clinic managers provided assent for their clinics to participate in the SAIA RCT. One staff member who participated in the SAIA intervention and one clinic manager from each FP clinic were recruited by our study team. Verbal assent to participate in the semi-structured interview was obtained from all participants at the time of the interview, witnessed by a second staff member, and documented by staff initials after completion of the survey. These processes were approved by the ethical review boards. Data were de-identified after collection. The interviews included a workplace assessment consisting of 53 and 45 questions for clinic staff and clinic managers, respectively. The surveys focused on the strategic organizational climate of FP clinics [See Additional File 1 and Additional File 2]. The questions were divided into organizational climate metrics and the ORIC 10-item assessment. The organizational climate survey consisted of eight metrics: management support (6 questions), relative priority (5 questions for clinic staff, 2 questions for clinic managers), commitment to facility (5 questions), upward communication (7 questions), tradition (4 questions), innovation and flexibility (6 questions), effort (5 questions), and supervisory support (5 questions, clinic staff only). The organizational climate survey included selected questions from the Organizational Citizenship and Behavior Checklist and Organizational Climate Measure, which have been shown to have high reliability and construct validity in other settings [24–26]. The questions from these two surveys were adapted from a previous SAIA trial in Mozambique by members of the research team [22]. The ORIC 10-item assessment consisted of domains for change commitment (5 questions) and change efficacy (5 questions) [15]. We hypothesized that domains of the organizational climate would contribute to change commitment and change efficacy, positively influencing HIV counseling and testing. Additionally, we hypothesized that elements of organizational climate could contribute directly to improvement in HIV counseling and testing (Fig 1).

This exploratory study utilized the sample from the parent trial, so there was not a separate sample size determination for this analysis.

The survey questions were presented as Likert-style items with response categories representing the respondents’ level of agreement with each statement. Based on earlier work in African clinical settings, the five-category Likert items were reduced to three categories, as respondents preferred differentiating between three levels of agreement [22]. These resulted in numerical scores of zero for ‘no’, one for ‘yes, a little’, and two for ‘yes, a lot’. By design, higher scores were applied to the more favorable response categories. The full survey is included as supplemental digital content.

Within each organizational climate metric, individual question scores were added together to generate a total score. For example, six questions for management support resulted in a score that ranged from 0-12. The ORIC 10-item assessment consisted of five questions about change readiness and five questions about change efficacy, each with a score range of 0–10.

Characteristics of clinics, staff members, and managers were summarized using proportions for binary and categorical variables and medians with interquartile range (IQR) for continuous variables. Scores for each organizational climate measure and ORIC domains were normalized to a proportion calculated as the number of points out of the total points for each category and multiplied by 100, with a maximum score of 100%. Median scores were then compared using the Wilcoxon Signed Rank test to compare manager/staff pairs within each facility. Primary analyses were performed using unadjusted linear regression to examine the association between organizational climate metrics (management support, relative priority, commitment to facility, upward communication, tradition, innovation and flexibility, effort, and supervisory support) as independent variables with change commitment and change efficacy as dependent variables. A secondary analysis was performed using the nonparametric Spearman’s correlation coefficient to compare organizational climate metrics with change commitment and change efficacy. We also examined organizational climate metrics, change commitment, and change efficacy as independent variables with the outcomes of percentage of clients counseled and percentage tested for HIV aggregated across the 12-month RCT for each FP clinic. Separate linear regression models were completed for clinic staff and clinic managers. Because of the small number of clinics, no multivariable modeling was performed. In addition, a secondary analysis was performed using Spearman’s correlation coefficient to compare organizational climate metrics, change commitment, and change efficacy with the percentage of clients counseled and tested for HIV. All analyses were conducted with SPSS. Reporting of these results abide by the Strengthening the Reporting of Observational Studies in Epidemiology (STROBE) guidelines [27].

## Results

### Demographics

Twelve clinics from the cluster-randomized trial were randomly assigned to the intervention arm. Following randomization, one clinic declined to participate in the interviews for the present analysis so 11 clinics were included in the analysis cohort for the study. In total, 11 clinic staff and 10 clinic managers completed the surveys (one clinic had no designated manager) between August 2, 2019 and September 26, 2019. Characteristics of the intervention clinics, staff, and managers are presented in Table 1. Six (55%) clinics were classified as public and five (45%) as private. Four (36%) clinics were urban, four (36%) were peri-urban, and three (27%) were rural. The clinics had a median of six staff members (IQR 1–9). The median number of trained HTC providers per clinic was one (interquartile range [IQR] 0–2). Nine (82%) clinic managers reported awareness of the Kenyan National HIV Testing and Counseling guidelines.

**Table 1:** Characteristics of 12 family planning clinics in Mombasa County, Kenya.

	N (%) Median (IQR)
**Public**	6 (55%)
**Private**	5 (45%)
**Urban**	4 (36%)
**Peri-urban**	4 (36%)
**Rural**	3 (27%)
**Payment required for family planning visits**	2 (18%)
**Number of clinic staff**	6 (1-9)
**Number of trained HTC providers**	1 (0-2)
**Awareness of Kenyan National HIV Testing and Counselling Guidelines**	9 (82%)
**Highest education level of clinic manager**	
Post-secondary certificate	1 (9%)
Post-secondary diploma	9 (82%)
Post-secondary higher diploma	1 (9%)

Abbreviations: IQR=interquartile range; FP=family planning; HIV=human immunodeficiency virus; HTC=HIV testing and counseling.

### Clinic staff and manager organizational readiness scores

The median organizational climate and ORIC domain scores for clinic staff and clinic managers are presented in Table 2. The median clinic staff score for relative priority of HTC was higher than that for clinic managers (0.90 (0.90, 0.90) vs. 0.75 (0.50, 0.75), p < 0.001) suggesting that clinic staff found implementing HTC to be a higher priority compared to clinic managers. Organizational climate measures and ORIC domain scores were otherwise similar for clinic staff and clinic managers.

**Table 2:** Median (IQR) scores for clinic staff and clinic managers.

Organizational Climate Measure	Clinic Manager Median (IQR), n = 10	Clinic Staff Median (IQR), n = 11	p-value
Management Support	0.83 (0.67, 0.83)	0.83 (0.81, 0.83)	0.2
Relative Priority	0.75 (0.50, 0.75)	0.90 (0.90, 0.90)	<0.001
Commitment to Facility	1.00 (1.00, 1.00)	1.00 (0.88, 1.00)	0.1
Upward Communication	0.71 (0.64, 0.71)	0.71 (0.57, 0.79)	0.6
Tradition	0.63 (0.63, 0.75)	0.63 (0.38, 0.78)	0.1
Innovation and Flexibility	1.00 (0.94, 1.00)	1.00 (0.83, 1.00)	0.3
Effort	0.65 (0.60, 0.78)	0.70 (0.60, 0.80)	0.2
Supervisory Support	--	1.00 (0.98, 1.00)	--
**ORIC Domain**			
Change Commitment	1.00 (1.00, 1.00)	1.00 (0.90, 1.00)	0.7
Change Efficacy	1.00 (1.00, 1.00)	1.00 (1.00, 1.00)	0.7

### Association between organizational climate measures and ORIC domains

The results of linear regression evaluating the relationship between clinic staff members’ and managers’ scores for organizational climate metrics and the ORIC domains are presented in Table 3. For clinic staff, higher scores for innovation and flexibility were associated with higher scores for change commitment (β = 0.20, 95% confidence interval [CI] 0.09-0.31, p = 0.001) and change efficacy (β = 0.17, 95% CI 0.07-0.26, p = 0.002). These results can be interpreted as showing that for each 1% increase in the innovation and flexibility score, there was a 0.2% increase in the score for change commitment and a 0.17% increase in the score for change efficacy. The results from the secondary analyses using Spearman’s correlation coefficient are also included in Table A in S1 Table. While there were differences in statistical significance for some of the associations for clinic managers, correlation coefficients are in the same direction as the beta coefficients in Table 3.

**Table 3:** Association between organizational climate measures and ORIC domains (change commitment, change efficacy).

Adapted Survey Metric	Beta	95% Confidence Intervals	p-value
**Clinic staff: Organizational climate measure score and change commitment**			
Management Support	-0.08	-0.26, 0.10	0.4
Relative Priority	-0.18	-0.54, 0.18	0.3
Commitment to Facility	0.03	-0.09, 0.15	0.6
Upward Communication	-0.05	-0.22, 0.12	0.6
Tradition	-0.06	-0.14, 0.03	0.2
Innovation and Flexibility	0.20	0.09, 0.31	0.001
Effort	-0.04	-0.25, 0.18	0.7
Supervisory Support	0.02	-0.13, 0.17	0.8
**Clinic staff: Organizational climate measure score and change efficacy**			
Management Support	-0.03	-0.19, 0.13	0.7
Relative Priority	0.12	-0.20, 0.43	0.4
Commitment to Facility	0.04	-0.06, 0.15	0.4
Upward Communication	-0.01	-0.16, 0.14	0.9
Tradition	0.02	-0.06, 0.10	0.6
Innovation and Flexibility	0.17	0.07, 0.26	0.002
Effort	0.10	-0.07, 0.28	0.2
Supervisory Support	-0.05	-0.18, 0.08	0.4
**Clinic managers: Organizational climate measure score and change commitment**			
Management Support	0.25	0.06, 0.45	0.01
Relative Priority	0.24	0.08, 0.39	0.004
Commitment to Facility	0.78	0.60, 0.96	<0.001
Upward Communication	-0.22	-0.82, 0.38	0.4
Tradition	-0.32	-0.65, 0.00	0.05
Innovation and Flexibility	0.44	0.04, 0.84	0.03
Effort	0.09	-0.25, 0.42	0.6
Supervisory Support	NA	NA	NA
**Clinic managers: Organizational climate measure score and change efficacy**			
Management Support	0.36	0.16, 0.56	0.001
Relative Priority	0.33	0.18, 0.48	<0.001
Commitment to Facility	0.60	0.21, 0.99	0.005
Upward Communication	-0.31	-1.00, 0.38	0.4
Tradition	-0.38	-0.75, -0.01	0.05
Innovation and Flexibility	0.33	-0.17, 0.82	0.2
Effort	-0.10	-0.48, 0.28	0.6
Supervisory Support	NA	NA	NA

Abbreviations: NA = not applicable.

For clinic managers, four organizational climate metric scores were positively associated with change commitment scores: management support (β = 0.25, 95% CI 0.06-0.45, p = 0.01), relative priority (β = 0.24, 95% CI 0.08-0.39, p = 0.004), commitment to facility (β = 0.78, 95% CI 0.60-0.96, p = 0.001), and innovation and flexibility (β = 0.44, 95% CI 0.04-0.84, p = 0.03). Three metrics were positively associated with higher change efficacy scores: management support (β = 0.36, 95% CI 0.16-0.56, p = 0.001), relative priority (β = 0.33, 95% CI 0.18-0.48, p < 0.001), and commitment to facility (β = 0.60, 95% CI 0.21-0.99, p = 0.005). Conversely, the organizational climate score for tradition was negatively associated with change efficacy (β = -0.38, 95% CI -0.75--0.01, p = 0.05).

### Association between organizational climate, organizational readiness and HTC

The results of linear regression evaluating the relationship between clinic staff members’ scores on the organizational climate metrics, ORIC domains, and the proportion of new FP clients counseled for HIV testing are presented in Table 4. The organizational climate metric score for management support was positively associated with the proportion of clients counseled for HIV testing (β = 1.20; 95% CI 0.08-2.32; p = 0.04). This can be interpreted as showing that for each 1% increase in the management support score, there was a predicted 1.2% higher proportion of new FP clients who received pre-HIV test counseling. Other metrics including relative priority, supervisory support, commitment to facility, upward communication, tradition, innovation and flexibility, and effort were not significantly associated with proportion of clients counseled. The ORIC domains of change commitment and change efficacy were also not associated with the proportion of FP clients counseled. There were no associations between organizational climate metrics or ORIC domains and the proportion of FP clients tested for HIV. The results from the secondary analyses using Spearman’s correlation coefficient are included in Table B in S1 Table. The correlation coefficients were of similar direction of association as the linear regression results, though the one statistically significant result for management support by linear regression was not statistically significant by nonparametric correlation coefficient.

**Table 4:** Association between organizational climate metrics, ORIC domains (change commitment, change efficacy), and FP client HTC.

Survey Metric	Beta	95% Confidence Intervals	p-value
**Clinic staff: Clients counseled on HIV testing**			
Management Support	1.20	0.08, 2.32	0.04
Relative Priority	1.37	-1.07, 3.80	0.3
Commitment to Facility	0.07	-0.77, 0.91	0.9
Upward Communication	-0.28	-1.46, 0.90	0.6
Tradition	-0.13	-0.73, 0.48	0.7
Innovation and Flexibility	0.00	-0.97, 0.97	1.0
Effort	0.24	-1.21, 1.68	0.7
Supervisory Support	0.58	-0.42, 1.57	0.2
Change Commitment	0.11	-3.07, 3.28	0.9
Change Efficacy	-0.86	-4.51, 2.78	0.6
**Clinic staff: Clients tested for HIV**			
Management Support	0.71	-0.31, 1.73	0.2
Relative Priority	0.36	-1.78, 2.49	0.7
Commitment to Facility	-0.18	-0.89, 0.53	0.6
Upward Communication	0.15	-0.86, 1.16	0.8
Tradition	-0.02	-0.54, 0.50	0.9
Innovation and Flexibility	-0.17	-1.00, 0.65	0.7
Effort	0.40	-0.82, 1.62	0.5
Supervisory Support	0.21	-0.66, 1.08	0.6
Change Commitment	0.36	-2.35, 3.06	0.8
Change Efficacy	-1.38	-4.44, 1.68	0.4
**Clinic Managers: Clients counseled on HIV Testing**			
Management Support	0.47	-0.43, 1.37	0.3
Relative Priority	-0.15	-0.92, 0.61	0.7
Commitment to Facility	-0.36	-2.03, 1.32	0.7
Upward Communication	1.71	-0.56, 3.97	0.1
Tradition	0.92	-0.45, 2.28	0.2
Innovation and Flexibility	0.49	-1.29, 2.27	0.6
Effort	0.46	-0.84, 1.76	0.5
Supervisory Support	NA	NA	NA
Change Commitment	-0.09	-2.05, 1.87	0.9
Change Efficacy	-0.56	-2.24, 1.12	0.5
**Clinic Managers: Clients tested for HIV**			
Management Support	0.44	-0.27, 1.15	0.2
Relative Priority	0.31	-0.28, 0.91	0.3
Commitment to Facility	-0.10	-1.45, 1.26	0.9
Upward Communication	-0.13	-2.07, 1.82	0.9
Tradition	0.45	-0.69, 1.58	0.4
Innovation and Flexibility	-0.11	-1.55, 1.34	0.9
Effort	-0.33	-1.37, 0.72	0.5
Supervisory Support	NA	NA	NA
Change Commitment	0.58	-0.97, 2.13	0.4
Change Efficacy	0.40	-0.96, 1.75	0.5

Abbreviations: NA = not applicable.

For clinic managers, there was a statistical trend suggesting a modest association between upward communication (β = 1.71, 95% CI -0.56-3.97, p = 0.1) and the proportion of FP clients counseled for HIV testing, though confidence intervals were wide (Table 4). All other organizational climate metrics, as well as the ORIC domains of change commitment and change efficacy, were not associated with clients counseled or tested for HIV.

## Discussion

In this study of Kenyan FP clinics testing the SAIA implementation strategy to optimize HTC, innovation and flexibility were predictive of organizational readiness to implement HTC for both clinic staff and clinic managers. In addition, clinic managers’ commitment to facility was strongly associated with organizational readiness. Although the small number of clinics in the study limited power for analyses of client outcomes, there was a strong association between staff members’ perception of management support and performance of more pre-HIV test counseling, one of the primary trial outcomes.

The relationships observed in this study showing associations between the organizational characteristics of management support, commitment to facility, and innovation and flexibility, and the ORIC domains of change commitment and change efficacy, support the concept that a culture with supportive leadership, readiness to accept and incorporate new ideas, and committed staff are useful indicators of an organization’s readiness for change [28,29]. For clinic managers, the organizational characteristic of tradition was associated with lower change efficacy scores and trended towards lower change commitment scores indicating that an environment that values the status quo negatively impacts readiness for change. Interestingly, many of the relationships between organizational characteristics and readiness for change were much stronger for clinic managers than for clinic staff. Managers may feel more empowered to enact changes compared to clinic staff, leading to stronger associations with organizational readiness.

In this study, staff perceptions of greater management support were associated with higher proportions of FP clients receiving HIV counselling. These results reflect that higher perceived support from one’s supervisors may be an important predictor of success in implementing change. It is possible that staff perceptions of management support were not associated with higher proportions of clients tested for HIV because it requires the client to complete additional actions in contrast to HIV counseling which is at the discretion of the provider. Similarly, in the cluster randomized trial testing SAIA as an implementation strategy to increase HIV counseling and testing in FP clinics, intervention clinics achieved larger increases in HIV counseling than in HIV testing [13]. This may be because clinic staff have greater control over the counseling outcome, whereas factors like stock outs, convenience fees, or clients declining the HIV test may present additional barriers to completion of testing [30]. Manager perceptions of upward communication trended towards higher proportions of FP clients receiving HIV counselling, though confidence intervals were wide. Other studies have demonstrated that organizational metrics such as change commitment, change efficacy, available resources, and knowledge of tasks are predictors of successful change, but the present analysis had limited power to detect similar findings [31].

Results were in the same direction using linear regression and Spearman’s correlation coefficient for all of the results, though there were less statistically significant results using the nonparametric test. This could be due to the lower statistical power of the nonparametric test or some of the variables not meeting the assumption of Normality for linear regression. However, a combination of these two analytic techniques paints a similar picture in this exploratory analysis.

This study has several strengths. We had high quality data on the clinical outcomes of HIV counselling and testing from the cluster randomized trial using SAIA to implement HTC, which allowed us to examine associations between the ORIC domains, organizational climate metrics, and clinical outcomes [13]. In addition, the clinics included public, private, urban, peri-urban, and rural facilities, making the results more generalizable to diverse types of FP clinics.

The results of this exploratory study should be interpreted in the context of a number of limitations and without overstating our findings. First, the small sample size provided limited power and precision in many of the analyses. Second, the sample of clinics, staff, and managers may not be generalizable to all FP clinics across Mombasa County or Kenya. Despite these limitations, we were able to detect several potentially important associations between organizational climate and organizational readiness for change that can be used to help generate hypotheses for future research. Third, multiple hypothesis tests were performed, increasing the risk of chance associations (type I error), so the results should be interpreted with caution. Fourth, the ORIC tool was psychometrically validated in the US, but has not been validated for use in sub-Saharan Africa. However, we did utilize adaptations of the ORIC tool arising from our prior work in Mozambique. Fifth, the organizational climate measures and ORIC question surveys were collected during the second half of the intervention. This had the advantage of allowing us to explore commitment to the ongoing intervention but does not allow us to draw conclusions about how baseline characteristics may have been associated with HTC outcomes. Future studies could benefit from examining these measures at multiple time points including prior to the initiation of an intervention.

## Conclusion

Our results add to a small body of literature looking at the organizational climate metrics’ ability to predict implementation effectiveness [31]. While various studies have evaluated whether organizational characteristics can predict organizational readiness, it will be important to expand the body of research on whether organizational characteristics can predict clinical outcomes in healthcare settings. Our findings also join the existing evidence that support from leadership and innovation/flexibility are important predictors of readiness for change. Future studies should investigation whether a focus on these domains can improve implementation of evidence-based interventions. While this study was exploratory, it suggests a potential hypothesis that enhancing management support can increase readiness for change and the likelihood of successful implementation.

## Supporting information

S1 Text Clinic Staff Survey. (DOCX)

S2 Text Clinic Manager Survey. (DOCX)

S1 Table Supplementary Tables A and B. (DOCX)

S1 Data Clinic staff database. (XLSX)

S2 Data Clinic manager database. (XLSX)

## Abbreviations

HIV: Human Immunodeficiency Virus
HTC: HIV testing and counselling
FP: Family planning
SAIA: Systems Analysis and Improvement Approach
ORIC: Organizational Readiness for Implementing Change
STROBE: Strengthening the Reporting of Observational Studies in Epidemiology
NA: Not applicable

## References

[1] UNAIDS. UNAIDS Data. 2023.

[2] De Cock KM, Barker JL, Baggaley R, El Sadr WM. Where are the positives? HIV testing in sub-Saharan Africa in the era of test and treat. AIDS. 2019;33(2):349–52. doi: 10.1097/QAD.0000000000002096 30557162

[3] Integration of HIV testing and linkage in family planning and contraception services: implementation brief. Geneva: World Health Organization; 2021.

[4] Narasimhan M, Yeh PT, Haberlen S, Warren CE, Kennedy CE. Integration of HIV testing services into family planning services: a systematic review. Reprod Health. 2019;16(Suppl 1):61. doi: 10.1186/s12978-019-0714-9 31138307 PMC6538541

[5] UNAIDS. Country fact sheets: Kenya. 2024.

[6] National AIDS/STD Control Programme MoPHaS. Guidelines for HIV testing and counselling in Kenya. Nairobi: NASCOP; 2010.

[7] National AIDS and STI Control Programme MoH, Kenya. Guidelines for HIV Testing Services in Kenya. Nairobi: NASCOP; 2015.

[8] Eastment MC, Wanje G, Richardson BA, Nassir F, Mwaringa E, Barnabas RV, et al. Performance of family planning clinics in conducting recommended HIV counseling and testing in Mombasa County, Kenya: a cross-sectional study. BMC Health Serv Res. 2019;19(1):665. doi: 10.1186/s12913-019-4519-x 31521157 PMC6744633

[9] Rustagi AS, Gimbel S, Nduati R, Cuembelo MdF, Wasserheit JN, Farquhar C, et al, with input from the SST. Implementation and Operational Research: Impact of a Systems Engineering Intervention on PMTCT Service Delivery in Côte d’Ivoire, Kenya, Mozambique: A Cluster Randomized Trial. J Acquir Immune Defic Syndr. 2016;72(3):e68-76. doi: 10.1097/QAI.0000000000001023 27082507 PMC4911259

[10] Wagner AD, Augusto O, Njuguna IN, Gaitho D, Mburu N, Oluoch G, et al. Systems Analysis and Improvement Approach to optimize the pediatric and adolescent HIV Cascade (SAIA-PEDS): a pilot study. Implement Sci Commun. 2022;3(1):49. doi: 10.1186/s43058-022-00272-8 35538591 PMC9087970

[11] Gimbel S, Mocumbi AO, Ásbjörnsdóttir K, Coutinho J, Andela L, Cebola B, et al. Systems analysis and improvement approach to optimize the hypertension diagnosis and care cascade for PLHIV individuals (SAIA-HTN): a hybrid type III cluster randomized trial. Implement Sci. 2020;15(1):15. doi: 10.1186/s13012-020-0973-4 32143657 PMC7059349

[12] Sherr K, Gimbel S, Rustagi A, Nduati R, Cuembelo F, Farquhar C, et al. Systems analysis and improvement to optimize pMTCT (SAIA): a cluster randomized trial. Implement Sci. 2014;9:55. doi: 10.1186/1748-5908-9-55 24885976 PMC4019370

[13] Eastment MC, Wanje G, Richardson BA, Mwaringa E, Sherr K, Barnabas RV, et al. Results of a cluster randomized trial testing the systems analysis and improvement approach to increase HIV testing in family planning clinics. AIDS. 2022;36(2):225–35. doi: 10.1097/QAD.0000000000003099 34628439 PMC8702477

[14] Weiner BJ, Amick H, Lee S-YD. Conceptualization and measurement of organizational readiness for change: a review of the literature in health services research and other fields. Med Care Res Rev. 2008;65(4):379–436. doi: 10.1177/1077558708317802 18511812

[15] Weiner BJ. A theory of organizational readiness for change. Implement Sci. 2009;4:67. doi: 10.1186/1748-5908-4-67 19840381 PMC2770024

[16] Shea CM, Jacobs SR, Esserman DA, Bruce K, Weiner BJ. Organizational readiness for implementing change: a psychometric assessment of a new measure. Implement Sci. 2014;9:7. doi: 10.1186/1748-5908-9-7 24410955 PMC3904699

[17] Sharma N, Herrnschmidt J, Claes V, Bachnick S, De Geest S, Simon M, et al. Organizational readiness for implementing change in acute care hospitals: An analysis of a cross-sectional, multicentre study. J Adv Nurs. 2018;74(12):2798–808. doi: 10.1111/jan.13801 30019540

[18] Randall CL, Hort K, Huebner CE, Mallott E, Mancl L, Milgrom P, et al. Organizational Readiness to Implement System Changes in an Alaskan Tribal Dental Care Organization. JDR Clin Trans Res. 2020;5(2):156–65. doi: 10.1177/2380084419871904 31499017 PMC7079331

[19] Helfrich CD, Kohn MJ, Stapleton A, Allen CL, Hammerback KE, Chan KCG, et al. Readiness to Change Over Time: Change Commitment and Change Efficacy in a Workplace Health-Promotion Trial. Front Public Health. 2018;6:110. doi: 10.3389/fpubh.2018.00110 29740572 PMC5925216

[20] Batterham P, Allenhof C, Cerga Pashoja A, Etzelmueller A, Fanaj N, Finch T, et al. Psychometric properties of two implementation measures: Normalization MeAsure Development questionnaire (NoMAD) and organizational readiness for implementing change (ORIC). Implement Res Pract. 2024;5:26334895241245448. doi: 10.1177/26334895241245448 38686322 PMC11057218

[21] A Steen J, Stewart C. A Psychometric Analysis of the Organizational Readiness for Implementing Change (ORIC) in a Child Welfare Setting. J Evid Based Soc Work (2019). 2024;21(6):704–19. doi: 10.1080/26408066.2024.2409092 39350343

[22] Sherr K, Ásbjörnsdóttir K, Crocker J, Coutinho J, de Fatima Cuembelo M, Tavede E, et al. Scaling-up the Systems Analysis and Improvement Approach for prevention of mother-to-child HIV transmission in Mozambique (SAIA-SCALE): a stepped-wedge cluster randomized trial. Implement Sci. 2019;14(1):41. doi: 10.1186/s13012-019-0889-z 31029171 PMC6487047

[23] Leslie HH, West R, Twine R, Masilela N, Steward WT, Kahn K, et al. Measuring Organizational Readiness for Implementing Change in Primary Care Facilities in Rural Bushbuckridge, South Africa. Int J Health Policy Manag. 2022;11(7):912–8. doi: 10.34172/ijhpm.2020.223 33300775 PMC9808169

[24] Fox S S, PE. Organizational Citizenship Behavior Checklist. 2009 [cited 2025 October 28]. Available from: https://paulspector.com/assessments/pauls-no-cost-assessments/organizational-citizenship-behavior-checklist-ocb-c/

[25] Patterson MG, West MA, Shackleton VJ, Dawson JF, Lawthom R, Maitlis S, et al. Validating the organizational climate measure: links to managerial practices, productivity and innovation. J Organ Behavior. 2005;26(4):379–408. doi: 10.1002/job.312

[26] Neves PC, Palma-Moreira A, Andrade C, Au-Yong-Oliveira M. Organizational citizenship behavior: adaptation and validation of the OCB scale CCOE-R. Front Psychol. 2024;15:1475011. doi: 10.3389/fpsyg.2024.1475011 39764075 PMC11701931

[27] von Elm E, Altman DG, Egger M, Pocock SJ, Gøtzsche PC, Vandenbroucke JP, et al. The Strengthening the Reporting of Observational Studies in Epidemiology (STROBE) statement: guidelines for reporting observational studies. Lancet. 2007;370(9596):1453–7. doi: 10.1016/S0140-6736(07)61602-X 18064739

[28] von Treuer K, Karantzas G, McCabe M, Mellor D, Konis A, Davison TE, et al. Organizational factors associated with readiness for change in residential aged care settings. BMC Health Serv Res. 2018;18(1):77. doi: 10.1186/s12913-018-2832-4 29390999 PMC5796299

[29] Claiborne N, Auerbach C, Lawrence C, Schudrich WZ. Organizational change: The role of climate and job satisfaction in child welfare workers’ perception of readiness for change. Children Youth Serv Rev. 2013;35(12):2013–9. doi: 10.1016/j.childyouth.2013.09.012

[30] Long JE, Eastment MC, Wanje G, Richardson BA, Mwaringa E, Mohamed MA, et al. Assessing the sustainability of the Systems Analysis and Improvement Approach to increase HIV testing in family planning clinics in Mombasa, Kenya: results of a cluster randomized trial. Implement Sci. 2022;17(1):70. doi: 10.1186/s13012-022-01242-3 36195890 PMC9530422

[31] Nitturi V, Chen T-A, Kyburz B, Martinez Leal I, Correa-Fernandez V, O’Connor DP, et al. Organizational Characteristics and Readiness for Tobacco-Free Workplace Program Implementation Moderates Changes in Clinician’s Delivery of Smoking Interventions within Behavioral Health Treatment Clinics. Nicotine Tob Res. 2021;23(2):310–9. doi: 10.1093/ntr/ntaa163 32832980 PMC7822101

