# Supplementary material for: Cross-sectional study evaluating organizational climate, change commitment, and change efficacy for predicting family planning clinics’ success in increasing HIV counseling and testing in Mombasa, Kenya
Source: PLOS Glob Public Health. 2025 Dec 31;5(12):e0005542. doi: 10.1371/journal.pgph.0005542 (PMC12755772; doi:10.1371/journal.pgph.0005542)
Supplement: S1 Text — (DOCX) [file pgph.0005542.s001.docx]

Additional File 1: Clinic Staff ORIC Case Report Form (CRF)

Date of interview: _____________________ (day/month/year)

Position of staff being interviewed: ________________________

FP Clinic number:_____________________________________________

**Interviewer prompt: I am going to ask you a series of questions about the implementation of HIV testing for new family planning clinic clients. I will ask you to respond to the questions using one of three categories: no, yes—a little, or yes—strongly.**

**Management Support for increasing HIV testing in family planning clinic clients**

| # | Question | Responses |
| --- | --- | --- |
| 1 | Are the supervisors committed to the successful implementation of increasing HIV testing in family planning clinic clients?  0= No  If yes, how committed are the supervisors to successfully implementing the intervention?  1= A little committed  2= Strongly committed |  |
| 2 | Have the supervisors expressed doubts about whether increasing HIV testing in family planning clinic clients will help this facility?  0= No  If yes, how much doubt have they expressed?  1= A little doubt about the intervention  2=Strong doubts about the intervention |  |
| 3 | Do the supervisors show interest in increasing HIV testing in family planning clinic clients?  0= No  If yes, how much interest do supervisors show?  1= A little interest  2= Strong interest |  |
| 4 | Do the supervisors stress the importance of increasing HIV testing in family planning clinic clients at this facility?  0= No  If yes, how much do they stress the importance of the intervention?  1= Supervisors stress the importance of the intervention a little  2= Supervisors stress the importance of the intervention a lot |  |

| 5 | Do supervisors take an active interest in increasing HIV testing in family planning clinic clients’ successes and problems?  0= No  If yes, how much of an active interest in increasing HIV testing in family planning clinic clients’ successes and problems do they take?  1= A little interest  2= A lot of interest |  |
| --- | --- | --- |
| 6 | Do the supervisors at this facility make an effort to ensure increasing HIV testing in family planning clinic clients is a success here?  0= No  If yes, how much effort are the supervisors making?  1= A little effort  2= A lot of effort |  |

**Relative Priority**

| # | Question | Responses |
| --- | --- | --- |
| 7 | Is increasing HIV testing in family planning clinic clients a priority at this clinic?  0= No  If yes, how much of a priority is increasing HIV testing in family planning clinic clients at this clinic?  1= A small priority  2= A big priority |  |
| 8 | Do other projects take priority over increasing HIV testing in family planning clinic clients at this clinic?  0= No  If yes, how much of a priority do other projects take over increasing HIV testing in family planning clinic clients at this clinic?  1= A small priority  2= A big priority |  |
| 9 | Does facility staff put in effort into making increasing HIV testing in family planning clinic clients a success here?  0= No  If yes, how much effort does facility staff take to make increasing HIV testing in family planning clinic clients a success here?  1= A little effort  2= A lot of effort |  |
| 10 | Does facility staff think that the implementation of increasing HIV testing in family planning clinic clients is important?  0= No  If yes, how important is the implementation of increasing HIV testing in family planning clinic clients to facility staf?  1= A little important  2= Very important |  |
| 11 | Does facility staff think that the effective use of increasing HIV testing in family planning clinic clients is important?  0= No  If yes, how important is the effective use of increasing HIV testing in family planning clinic clients to facility staff ?  1= A little important  2= Very important |  |

**Commitment to the Facility**

| # | Questions | Responses |
| --- | --- | --- |
| 12 | Are people here willing to put in effort beyond what is normally expected in order to help this clinic be successful?  0= No  If yes, how willing are people to put in effort beyond what is normally expected in order to help?  1= A little willing to put in effort  2= Very willing to put in effort |  |
| 13 | Do employees state that this clinic is a great place to work?  0= No  If yes, how often do employees state this clinic is a great place to work?  1= A little  2= A lot |  |
| 14 | Does this clinic inspire employees to perform their best at their job?  0= No  If yes, how much does the clinic inspire employees to perform their best?  1= The clinic inspires them a little  2= The clinic inspires them a lot |  |
| 15 | Do people here really care about the fate of this clinic?  0= No  If yes, how much do people care about the fate of this clinic?  1= People care about the fate of this clinic a little  2= People care a lot about the fate of this clinic a lot |  |
| 16 | Do people here feel loyal to this clinic?  0= No  If yes, how loyal do people feel to this clinic?  1= A little loyal  2= Very loyal |  |

**Upward Communication**

| # | Questions | Responses |
| --- | --- | --- |
| 17 | Do employees feel confident that their suggestions for clinic improvements are seriously considered by supervisors?  0= No  If yes, how confident do employees feel?  1= A little confident  2= Very confident |  |
| 18 | Do employees speak up when they disagree with a decision?  0= No  If yes, how much do employees speak up when they disagree with a decision?  1= A little  2= A lot |  |
| 19 | When employees here make suggestions to supervisors, do the supervisors take those suggestions seriously?  0= No  If yes, how seriously do the supervisors take these suggestions?  1= A little seriously  2= Very seriously |  |
| 20 | If employees disagree with something that is happening in this clinic, do they tell their supervisors?  0= No  If yes, how much do employees tell their supervisors that they disagree with something that is happening in this facility?  1= A little bit  2= A lot |  |
| 21 | Do employees avoid making suggestions for clinic improvements because they believe their suggestions will be ignored?  0= No  If yes, how much do employees avoid making suggestions for clinic improvements because they believe their suggestions will be ignored?  1= A little bit  2= A lot |  |
| 22 | Do supervisors usually ignore employee suggestions for improving the clinic?  0= No  If yes, how much do supervisors usually ignore employee suggestions for improving the clinic?  1= A little bit  2= A lot |  |
| 23 | Do employees talk to their supervisors about problems in the clinic?  0= No  If yes, how much do employees talk to their supervisors about problems in the clinic?  1= A little bit  2= A lot |  |

**Tradition (Culture)**

| # | Questions | Responses |
| --- | --- | --- |
| 24 | Does senior management like to keep to established, traditional ways of doing things?  0= No  If yes, how much does senior management like keep to established, traditional ways of doing things?  1= A little bit  2= A lot |  |
| 25 | Has the clinic ever changed the way it’s done things?  0= No  If yes, how much has this clinic changed the way it does things?  1= A little change  2= A lot of change |  |
| 26 | Is management interested in trying out new ideas?  0= No  If yes, how interested is management in trying out new ideas?  1= A little interest  2= A lot of interest |  |
| 27 | Do changes in the way things are going here happen very slowly?  0= No  If yes, how slowly does change happen here?  1= A little slowly  2= Very slowly |  |

**Innovation and Flexibility (Culture)**

| # | Questions | Responses |
| --- | --- | --- |
| 28 | Are new ideas readily accepted here?  0= No  If yes, how readily accepted are new ideas?  1= A little  2= A lot |  |
| 29 | Is this clinic quick to respond when changes need to be made?  0= No  If yes, how quickly does this clinic respond to changes that need to be made?  1= A little quick to respond  2= Very quick to respond |  |
| 30 | Is management at this facility quick to identify when things should be done differently?  0= No  If yes, how quickly does management identify when things should be done differently?  1= A little quickly  2= Very quickly |  |
| 31 | Is this clinic flexible? By flexible, we mean the speed in which the clinic can change procedures to meet new conditions and solve problems as they arise.  0= No  If yes, how flexible is this clinic?  1= A little bit flexible  2= Very flexible |  |
| 32 | Does the clinc provide assistance for developing new ideas?  0= No  If yes, how much assistance does the clinic provide?  1= A little assistance  2= A lot of assistance |  |
| 33 | Do people in this clinic search for new ways to look at a problem?  0= No  If yes, how much do people in this clinic search for new ways to look at a problem?  1= A little  2= A lot |  |

**Effort (Culture/Climate)**

| # | Questions | Responses |
| --- | --- | --- |
| 34 | Do people want to perform to the best of their ability?  0= No  If yes, how much do people want to perform to the best of their availability?  1= A little  2= A lot |  |
| 35 | Are people enthusiastic about their work?  0= No  If yes, how enthusiastic are they?  1= A little enthusiastic  2= Very enthusiastic |  |

| 36 | Do people here get by with doing as little as possible?  0= No  If yes, how often do people here do as little as possible to get by?  1= Not often at all  2= Very often |  |
| --- | --- | --- |
| 37 | Are people here prepared to make a special effort to do a good job?  0= No  If yes, how prepared are they?  1= A little prepared  2= Very prepared |  |
| 38 | Do people here avoid putting more effort into their work than they have to?  0= No  If yes, how much do people here avoid putting more effort into their work than they have to?  1= A little  2= A lot |  |

Supervisory Support (Culture/Climate)

| # | Questions | Responses |
| --- | --- | --- |
| 39 | Are supervisors here good at understanding other people’s problems?  0= No  If yes, how good are supervisors at understanding people’s problems?  1= A little good  2= Very good |  |
| 40 | Do supervisors have confidence in those they manage?  0= No  If yes, how much confidence do supervisors have in those they manage?  1= A little  2= A lot |  |

| 41 | Are supervisors here friendly and easy to approach?  0= No  If yes, how friendly and easy to approach are the supervisors?  1= A little friendly and easy to approach  2= Very friendly and easy to approach |  |
| --- | --- | --- |
| 42 | Can supervisors be relied upon to give good guidance to their employees?  0= No  If yes, how relied upon can supervisors be?  1= A little relied upon to give good guidance their employees  2= Very relied upon to give good guidance their employees |  |
| 43 | Do supervisors understand the people who work for them?  0= No  If yes, how much do supervisors understand the people who work for them?  1= Supervisors understand the people who work for them a little  2= Supervisors understand the people who work for them a lot |  |

ORIC Questions

| # | Questions | Responses |
| --- | --- | --- |
| 44 | Are the people who work here committed to implementing increasing HIV testing in family planning clinic clients?  0= No  If yes, how committed are the people who work here to implementing increasing HIV testing in family planning clinic clients?  1= A little committed  2= Very committed |  |

| 45 | Are the people who work here confident that they can keep track of progress in implementing increasing HIV testing in family planning clinic clients?  0= No  If yes, how confident are the people who work here that they can keep track of progress in implementing increasing HIV testing in family planning clinic clients?  1= A little confident  2= Very confident |  |
| --- | --- | --- |
| 46 | Will the people who work here will do whatever it takes to implement increasing HIV testing in family planning clinic clients?  0= No  If yes, how much will the people who work here do to implement increasing HIV testing in family planning clinic clients?  1= A little  2= A lot |  |
| 47 | Do the people who work here feel confident that the clinic can support people as they adjust to increasing HIV testing in family planning clinic clients?  0= No  If yes, how confident do the people who work here feel confident that the clinc can support people as they adjust to this change?  1= A little  2= A lot |  |
| 48 | Do the people who work here want to implement increasing HIV testing in family planning clinic clients?  0= No  If yes, how much do people who work here want to implement increasing HIV testing in family planning clinic clients?  1= A little  2= A lot |  |

| 49 | Do the people who work here feel confident that they can handle the challenges that might arise in implementing increasing HIV testing in family planning clinic clients?  0= No  If yes, how confident do the people who work here feel that they can handle the challenges that might arise in implementing increasing HIV testing in family planning clinic clients?  1= A little confident  2= Very confident |  |
| --- | --- | --- |
| 50 | Are people who work here are determined to implement increasing HIV testing in family planning clinic clients?  0= No  If yes, how determined are people who work here to implement increasing HIV testing in family planning clinic clients?  1= A little determined  2= Very determined |  |
| 51 | Do people who work here feel confident that they can coordinate tasks so that implementation goes smoothly?  0= No  If yes, how confident do people who work here feel that they can coordinate tasks so that implementation goes smoothly?  1= A little confident  2= Very confident |  |
| 52 | Are people who work here motivated to implement increasing HIV testing in family planning clinic clients?  0= No  If yes, how motivated are people who work here to implement increasing HIV testing in family planning clinic clients?  1= A little motivated  2= Very motivated |  |
| 53 | Do people who work here feel confident that they can manage the politics of implementing increasing HIV testing in family planning clinic clients?  0= No  If yes, how confident do people who work here feel that they can manage the politics of implementing increasing HIV testing in family planning clinic clients?  1= A little confident  2=Very confident |  |

Initials of person completing this CRF: ___________________

**References**

1. Shea CM, Jacobs SR, Esserman DA, Bruce K, Weiner BJ. Organizational readiness for implementing change: a psychometric assessment of a new measure. Implement Sci. 2014;9:7.
2. Fox S, Spector, PE. Organizational Citizenship Behavior Checklist [Internet]. Paul Spector; 2009 [cited 2023 May 03]. Available from: https://paulspector.com/assessments/pauls-no-cost-assessments/organizational-citizenship-behavior-checklist-ocb-c/.
3. Patterson MG, West MA, Shackleton VJ, Dawson JF, Lawthom R, Maitlis S, et al. Validating the organizational climate measure: links to managerial practices, productivity and innovation. Journal of Organizational Behavior. 2005;26(4):379-408.
