# Supplementary material for: Cross-sectional study evaluating organizational climate, change commitment, and change efficacy for predicting family planning clinics’ success in increasing HIV counseling and testing in Mombasa, Kenya
Source: PLOS Glob Public Health. 2025 Dec 31;5(12):e0005542. doi: 10.1371/journal.pgph.0005542 (PMC12755772; doi:10.1371/journal.pgph.0005542)
Supplement: S2 Text — (DOCX) [file pgph.0005542.s002.docx]

Additional File 2: Clinic Manager ORIC Case Report Form (CRF)

Date of interview: _____________________ (day/month/year)

FP Clinic number:_____________________________________________

**Interviewer prompt:** I am going to ask you a series of questions about the implementation of HIV testing for new family planning clinic clients. I will ask you to respond to the questions using one of three categories: no, yes—a little, or yes—strongly.

**Management Support for increasing HIV testing in family planning clinic clients**

| # | Question | Responses |
| --- | --- | --- |
| 1 | Is the larger facility committed to the successful implementation of increasing HIV testing in family planning clinic clients?  0= No  If yes, how committed is the larger facility to successfully implementing this intervention?  1= A little committed  2= Strongly committed |  |
| 2 | Has the larger facility expressed doubts about whether increasing HIV testing in family planning clinic clients will help this facility?  0= No  If yes, how much doubt have they expressed?  1= A little doubt about the intervention  2= Strong doubts about the intervention |  |
| 3 | Does the larger facility show interest in increasing HIV testing in family planning clinic clients?  0= No  If yes, how much interest does the larger facility show?  1= A little interest  2= A lot of interest |  |
| 4 | Does the larger facility stress the importance of increasing HIV testing in family planning clinic clients at this facility?  0= No  If yes, how much do they stress the importance of the intervention?  1= Supervisors stress the importance of the intervention a little  2= Supervisors stress the importance of the intervention a lot |  |
| 5 | Does the larger facility take an active interest in increasing HIV testing in family planning clinic clients’ successes and problems?  0= No  If yes, how much of an active interest in this intervention’s successes and problems do they take?  1= A little interest  2= A lot of interest |  |
| 6 | Does the larger facility make an effort to ensure that increasing HIV testing in family planning clinic clients is a success here?  0= No  If yes, how much effort does the larger facility make?  1= A little effort  2= A lot of effort |  |

**Relative Priority**

| # | Questions | Responses |
| --- | --- | --- |
| 7 | Is increasing HIV testing in family planning clinic clients a priority at this clinic?  0= No  If yes, how much of a priority is increasing HIV testing in family planning clinic clients at this clinic?  1= A small priority  2= A big priority |  |
| 8 | Do other projects take priority over increasing HIV testing in family planning clinic clients at this clinic?  0= No  If yes, how much of a priority do other projects take over increasing HIV testing in family planning clinic clients at this clinic?  1= A small priority  2= A big priority  Which other projects take priority over increasing HIV testing in family planning clinic clients? | Free text: |

**Commitment to the Facility**

| ~~#~~ | Questions | Responses |
| --- | --- | --- |
| 9 | Are people here willing to put in effort beyond what is normally expected in order to help this facility be successful?  0= No  If yes, how willing are people to put in effort beyond what is normally expected in order to help?  1= A little willing to put in effort  2= Very willing to put in effort |  |
| 10 | Do employees state that this facility is a great place to work?  0= No  If yes, how much do employees state this facility is a great place to work?  1= A little  2= A lot |  |
| 11 | Does this facility inspire employees to perform their best at their job?  0= No  If yes, how much does the facility inspire employees to perform their best?  1= The facility inspires them a little  2= The facility inspires them a lot |  |
| 12 | Do people here really care about the fate of this facility?  0= No  If yes, how much do people care about the fate of this facility?  1= People care about the fate of this facility a little  2= People care a lot about the fate of this facility a lot |  |
| 13 | Do people here feel loyal to this facility?  0= No  If yes, how loyal do people feel to this facility?  1= A little loyal  2= Very loyal |  |

**Upward Communication**

| # | Questions | Responses |
| --- | --- | --- |
| 14 | Do clinic mangers feel confident that their suggestions for facility improvements are seriously considered by the larger facility?  0= No  If yes, how confident do clinic managers feel?  1= A little confident  2= Very confident |  |
| 15 | Do clinic managers speak up when they disagree with a decision?  0= No  If yes, how much do clinic managers speak up when they disagree with a decision?  1= A little  2= A lot |  |
| 16 | When clinic managers make suggestions to supervisors, do the supervisors take those suggestions seriously?  0= No  If yes, how seriously do the supervisors take these suggestions?  1= A little seriously  2= Very seriously |  |
| 17 | If clinic managers disagree with something that is happening in this facility, do they tell their supervisors?  0= No  If yes, how much do clinic managers tell their supervisors that they disagree with something that is happening in this facility?  1= A little bit  2= A lot |  |
| 18 | Do clinic managers avoid making suggestions for facility improvements because they believe their suggestions will be ignored?  0= No  If yes, how much do clinic managers avoid making suggestions for facility improvements because they believe their suggestions will be ignored?  1= A little bit  2= A lot |  |
| 19 | Does the larger facility usually ignore clinic manager suggestions for improving the facility?  0= No  If yes, how much does the larger facility usually ignore employee suggestions for improving the facility?  1= A little bit  2= A lot |  |
| 20 | Do clinic managers talk to their supervisors about problems in the facility?  0= No  If yes, how much do clinic managers talk to their supervisors about problems in the facility?  1= A little bit  2= A lot |  |

**Tradition (Culture)**

| # | Questions | Responses |
| --- | --- | --- |
| 21 | Does senior management like to keep to established, traditional ways of doing things?  0= No  If yes, how much does senior management like keep to established, traditional ways of doing things?  1= A little bit  2= A lot |  |
| 22 | Has the clinic ever changed the way its done things?  0= No  If yes, how much has this clinic changed the way it does things?  1= A little change  2= A lot of change |  |
| 23 | Is management interested in trying out new ideas?  0= No  If yes, how interested is management in trying out new ideas?  1= A little interested  2= Very interested |  |

| 24 | Do changes in the way things are going here happen very slowly?  0= No  If yes, how slowly does change happen here?  1= A little slowly  2= Very slowly |  |
| --- | --- | --- |

**Innovation and Flexibility (Culture)**

| # | Questions | Responses |
| --- | --- | --- |
| 25 | Are new ideas readily accepted here?  0= No  If yes, how readily accepted are new ideas?  1= A little  2= A lot |  |
| 26 | Is the larger facility quick to respond when changes need to be made?  0= No  If yes, how quickly does the larger facility respond to changes that need to be made?  1= A little quick to respond  2= Very quick to respond |  |
| 27 | Is the larger facility quick to identify when things should be done differently?  0= No  If yes, how quickly does the larger facility identify when things should be done differently?  1= A little quickly  2= Very quickly |  |
| 28 | Is this clinic flexible? By flexible, we mean the speed in which the clinic can change procedures to meet new conditions and solve problems as they arise.  0= No  If yes, how flexible is this clinic?  1= A little bit flexible  2= Very flexible |  |
| 29 | Does the larger facility provide assistance for developing new ideas?  0= No  If yes, how much assistance does the larger facility provide?  1= A little assistance  2= A lot of assistance |  |
| 30 | Do people in this clinic search for new ways to look at a problem?  0= No  If yes, how much do people in this clinic search for new ways to look at a problem?  1= A little  2= A lot |  |

**Effort (Culture/Climate)**

| # | Questions | Responses |
| --- | --- | --- |
| 31 | Do people here want to perform to the best of their ability?  0= No  If yes, how much do people here want to perform to the best of their availability?  1= A little  2= A lot |  |
| 32 | Are people here enthusiastic about their work?  0= No  If yes, how enthusiastic are they?  1= A little enthusiastic  2= Very enthusiastic |  |
| 33 | Do people here get by with doing as little as possible?  0= No  If yes, how often do people here do as little as possible to get by?  1= Not often at all  2= Very often |  |
| 34 | Are people here prepared to make a special effort to do a good job?  0= No  If yes, how prepared are they?  1= A little prepared  2= Very prepared |  |
| 35 | Do people here avoid putting more effort into their work than they have to?  0= No  If yes, how much do people here avoid putting more effort into their work than they have to?  1= A little  2= A lot |  |

**ORIC Questions**

| # | Questions | Responses |
| --- | --- | --- |
| 36 | Are the people who work here committed to implementing increasing HIV testing in family planning clinic clients?  0= No  If yes, how committed are the people who work here to implementing increasing HIV testing in family planning clinic clients?  1= A little committed  2= Very committed |  |
| 37 | Are the people who work here confident that they can keep track of progress in implementing increasing HIV testing in family planning clinic clients?  0= No  If yes, how confident are the people who work here that they can keep track of progress in implementing increasing HIV testing in family planning clinic clients?  1= A little confident  2= Very confident |  |
| 38 | Will the people who work here do whatever it takes to implement increasing HIV testing in family planning clinic clients?  0= No  If yes, how much will people who work here do to implement increasing HIV testing in family planning clinic clients?  1= a little  2= a lot |  |
| 39 | Do the people who work here feel confident that the organization can support people as they adjust to increasing HIV testing in family planning clinic clients?  0= No  If yes, how confident do the people who work here feel confident that the organization can support people as they adjust to increasing HIV testing in family planning clinic clients?  1= A little  2= A lot |  |
| 40 | Do the people who work here want to implement increasing HIV testing in family planning clinic clients?  0= No  If yes, how much do people who work here want to implement increasing HIV testing in family planning clinic clients?  1= A little  2= A lot |  |
| 41 | Do the people who work here feel confident that they can handle the challenges that might arise in implementing increasing HIV testing in family planning clinic clients?  0= No  If yes, how confident do the people who work here feel that they can handle the challenges that might arise in implementing increasing HIV testing in family planning clinic clients?  1= A little confident  2= Very confident |  |
| 42 | Are people who work here are determined to implement increasing HIV testing in family planning clinic clients?  0= No  If yes, how determined are people who work here to implement increasing HIV testing in family planning clinic clients?  1= A little determined  2= Very determined |  |
| 43 | Do people who work here feel confident that they can coordinate tasks so that implementation goes smoothly?  0= No  If yes, how confident do people who work here feel that they can coordinate tasks so that implementation goes smoothly?  1= A little confident  2= Very confident |  |
| 44 | Are people who work here motivated to implement increasing HIV testing in family planning clinic clients?  0= No  If yes, how motivated are people who work here to implement increasing HIV testing in family planning clinic clients?  1= A little motivated  2= Very motivated |  |
| 45 | Do people who work here feel confident that they can manage the politics of implementing increasing HIV testing in family planning clinic clients?  0= No  If yes, how confident do people who work here feel that they can manage the politics of implementing increasing HIV testing in family planning clinic clients?  1= A little confident  2=Very confident |  |

Initials of person completing this CRF: ___________________

**References**

1. Shea CM, Jacobs SR, Esserman DA, Bruce K, Weiner BJ. Organizational readiness for implementing change: a psychometric assessment of a new measure. Implement Sci. 2014;9:7.
2. Fox S, Spector, PE. Organizational Citizenship Behavior Checklist [Internet]. Paul Spector; 2009 [cited 2023 May 03]. Available from: https://paulspector.com/assessments/pauls-no-cost-assessments/organizational-citizenship-behavior-checklist-ocb-c/.
3. Patterson MG, West MA, Shackleton VJ, Dawson JF, Lawthom R, Maitlis S, et al. Validating the organizational climate measure: links to managerial practices, productivity and innovation. Journal of Organizational Behavior. 2005;26(4):379-408.
