# Supplementary material for: Cross-sectional study evaluating organizational climate, change commitment, and change efficacy for predicting family planning clinics’ success in increasing HIV counseling and testing in Mombasa, Kenya
Source: PLOS Glob Public Health. 2025 Dec 31;5(12):e0005542. doi: 10.1371/journal.pgph.0005542 (PMC12755772; doi:10.1371/journal.pgph.0005542)
Supplement: S1 Table — (DOCX) [file pgph.0005542.s003.docx]

Table A. Association between organizational climate measures and ORIC domains (change commitment, change efficacy) using Spearman’s correlation coefficient.

| **Adapted Survey Metric** | **Spearman CC** | **p-value** |
| --- | --- | --- |
| **Clinic staff: Organizational climate measure score and change commitment** | | |
| Management Support | -0.21 | 0.4 |
| Relative Priority | -0.25 | 0.3 |
| Commitment to Facility | 0.22 | 0.3 |
| Upward Communication | -0.06 | 0.8 |
| Tradition | -0.28 | 0.2 |
| Innovation and Flexibility | 0.57 | 0.005 |
| Effort | -0.05 | 0.8 |
| Supervisory Support | 0.15 | 0.5 |
| **Clinic staff: Organizational climate measure score and change efficacy** | | |
| Management Support | -0.04 | 0.9 |
| Relative Priority | 0.26 | 0.2 |
| Commitment to Facility | 0.23 | 0.3 |
| Upward Communication | -0.05 | 0.8 |
| Tradition | 0.16 | 0.5 |
| Innovation and Flexibility | 0.61 | 0.003 |
| Effort | 0.27 | 0.2 |
| Supervisory Support | -0.25 | 0.3 |
| **Clinic managers: Organizational climate measure score and change commitment** | | |
| Management Support | 0.33 | 0.2 |
| Relative Priority | 0.36 | 0.1 |
| Commitment to Facility | 0.68 | <0.001 |
| Upward Communication | -0.14 | 0.6 |
| Tradition | -0.33 | 0.2 |
| Innovation and Flexibility | 0.68 | <0.001 |
| Effort | -0.01 | 0.9 |
| Supervisory Support | NA | NA |
| **Clinic managers: Organizational climate measure score and change efficacy** | | |
| Management Support | 0.34 | 0.1 |
| Relative Priority | 0.37 | 0.1 |
| Commitment to Facility | 0.28 | 0.2 |
| Upward Communication | -0.07 | 0.8 |
| Tradition | -0.32 | 0.2 |
| Innovation and Flexibility | 0.68 | 0.001 |
| Effort | -0.15 | 0.5 |
| Supervisory Support | NA | NA |

Abbreviations: CC=correlation coefficient; NA=not applicable

Table B. Association between organizational climate metrics, ORIC domains (change commitment, change efficacy), and FP client HTC using Spearman’s correlation coefficient.

| **Survey Metric** | **Spearman CC** | **p-value** |
| --- | --- | --- |
| **Clinic staff: Clients counseled on HIV testing** | | |
| Management Support | 0.27 | 0.2 |
| Relative Priority | -0.01 | 1.0 |
| Commitment to Facility | 0.08 | 0.7 |
| Upward Communication | -0.10 | 0.7 |
| Tradition | -0.02 | 0.9 |
| Innovation and Flexibility | 0.06 | 0.8 |
| Effort | 0.21 | 0.3 |
| Supervisory Support | 0.25 | 0.3 |
| Change Commitment | 0.11 | 0.6 |
| Change Efficacy | -0.04 | 0.9 |
| **Clinic staff: Clients tested for HIV** | | |
| Management Support | 0.18 | 0.4 |
| Relative Priority | -0.11 | 0.6 |
| Commitment to Facility | -0.21 | 0.3 |
| Upward Communication | 0.08 | 0.7 |
| Tradition | -0.03 | 0.9 |
| Innovation and Flexibility | -0.03 | 0.9 |
| Effort | 0.09 | 0.7 |
| Supervisory Support | 0.33 | 0.1 |
| Change Commitment | 0.02 | 0.9 |
| Change Efficacy | -0.28 | 0.2 |
| **Clinic Managers: Clients counseled on HIV Testing** | | |
| Management Support | 0.32 | 0.2 |
| Relative Priority | 0.19 | 0.4 |
| Commitment to Facility | -0.22 | 0.3 |
| Upward Communication | 0.31 | 0.2 |
| Tradition | 0.40 | 0.08 |
| Innovation and Flexibility | 0.20 | 0.4 |
| Effort | 0.08 | 0.7 |
| Supervisory Support | NA | NA |
| Change Commitment | 0.13 | 0.6 |
| Change Efficacy | -0.005 | 1.0 |
| **Clinic Managers: Clients tested for HIV** | | |
| Management Support | 0.15 | 0.5 |
| Relative Priority | 0.32 | 0.2 |
| Commitment to Facility | -0.13 | 0.6 |
| Upward Communication | -0.03 | 0.9 |
| Tradition | 0.27 | 0.2 |
| Innovation and Flexibility | 0.04 | 0.9 |
| Effort | -0.21 | 0.4 |
| Supervisory Support | NA | NA |
| Change Commitment | 0.20 | 0.4 |
| Change Efficacy | 0.07 | 0.8 |

Abbreviations: CC=correlation coefficient; NA=not applicable
